# Correlation between sperm DNA fragmentation index and semen parameters in 418 men seen at a fertility center

**DOI:** 10.5935/1518-0557.20200079

**Published:** 2021

**Authors:** Láyonal Germán Acosta Campos, Lissett Chiscul Requejo, Carlos Antonio Rivas Miñano, Jheny Díaz Orrego, Elmer Chávez Loyaga, Luis Gonzales Cornejo

**Affiliations:** 1 IN VITRO GESTAR, Assisted Reproduction Center. Chiclayo, Perú

**Keywords:** Normozoospermia, Sperm DNA fragmentation, Infertile, progressive motility, oligoasthenoteratozoospermia

## Abstract

**Objective:**

The aim of this study was to determine the correlation between semen parameters and sperm DNA fragmentation index (DFI) in 418 men who attended a fertility center.

**Methods:**

This retrospective study includes 418 male patients seen at In Vitro Gestar, Chiclayo - Perú between October 2013 and March 2020. DFI was determined between normozoospermic patients (NORMO) and patients with one altered parameter (ALTERED). The patients were classified as oligozoospermic [Severe/Moderate oligozoospermia (OLIGO SM): <5x106 sperm/mL and Mild oligozoospermia (OLIGO M): 5 to <15x106 sperm/mL], asthenozoospermic [Severe asthenozoospermia (ASTHENO S): <10% PM (progressive motility); Moderate asthenozoospermia (ASTHENO MO): 10 to <20% PM; Mild asthenozoospermia (ASTHENO M): 20 to <32% PM)], teratozoospermic (TERATO) and oligoasthenoteratozoospermic (OAT). DFI was determined between these groups and NORMO. The data was analyzed using the SPSS 22.0 software for Windows.

**Results:**

Normozoospermic patients presented lower significant levels of DFI compared with all groups. NORMO (15.64±7.65) vs [ALTERED (18.41±9.43, *p*=0.003); OLIGO SM (26.38±12.94, *p*<0.005); ASTHENO S (23.09±11.45, *p*<0.01); TERATO (17.96±9.23, *p*<0.05) and OAT (22.05±12.15, *p*=0.001)]. We determined a significant negative correlation between DFI and progressive motility (r= -0.162 *p*=0.001), and those with normal morphology (r= -0.100 *p*=0.040). Likewise the DFI shows a significant positive correlation with age (r= 0.257 *p*=0.000).

**Conclusions:**

Our study establishes that high DFI is accompanied by a significant impairment in all seminal parameters.

## INTRODUCTION

Infertility is a disease of the reproductive system, defined by the failure to achieve a clinical pregnancy after 12 months or more of regular unprotected sexual intercourse (De Neubourg *et al*., 2012), and it affects approximately 8 to 12% of the word population (Kumar & Singh, 2015). Male factor is implicated in almost 50% of cases and out of these; approximately 30 - 40% are idiopathic (Kadioglu & Ortac, 2017). These facts determine the importance of semen evaluation to verify fertility potential.

Conventional semen analysis or spermogram (sperm concentration, motility, morphology and vitality) is used for male fertility evaluation (WHO, 2010). However, it is estimated that approximately 10-15% of infertile men present normal semen analysis (Sharma *et al*., 2004; Agarwal & Allamaneni, 2005; Lewis *et al*., 2008). Likewise, the spermogram does not measure the fertilizing potential of spermatozoa and the complex changes that occur in the female reproductive tract before fertilization (Wang & Swerdloff, 2014). Therefore, there may be other factors that affect male fertility and are not detected by the spermogram, for example: sperm membrane defects (Rajeev & Reddy, 2004), environmental factors (Oliva *et al*., 2001), genetic factors (Kim *et al*., 2017) and sperm DNA fragmentation (Quintero *et al*., 2015; Ioannou *et al*., 2016).

Recent studies indicate that sperm DNA fragmentation test is a useful diagnostic tool in male infertility evaluation (Agarwal *et al*., 2016). Intact sperm DNA is necessary for correct transmission of genetic material to the next generation. High percentage of sperm DNA fragmentation index (DFI) is associated with reduced fertilization rates, early development embryo, embryo quality, pregnancy rates and higher rates of spontaneous miscarriage (Bungum *et al*., 2007; Lewis *et al*., 2013; Simon *et al*., 2014). Sperm DNA fragmentation can be caused by many factors, such as: apoptosis during sperm maturation in the seminiferous tubule epithelium (Gosálvez *et al*., 2015), defects in chromatin packaging and remodeling during the process of spermiogenesis (Sakkas *et al*., 2002; Shamsi *et al*., 2008), increase of reactive oxygen species (ROS) (Aitken & De luliis, 2010; Alahmar, 2019) and decrease of seminal antioxidant (Shamsi *et al*., 2010). Obesity, stress, alcohol consumption, smoking, use of drugs, caffeine, diet and advanced age are also factors that increase DFI (Durairajanayagam, 2018).

The most common cause of DNA fragmentation in spermatozoa is reactive oxygen species (ROS) and oxidative stress (Agarwal *et al*., 2014; Majzoub *et al*., 2018; 2019). ROS are oxygen-derived free radicals, such as hydroxyl radicals (OH), superoxide anion (O_2_^-^) and hydrogen peroxide (H_2_O_2_). Low levels of ROS are required for sperm capacitation, hyperactivation, acrosome reaction and spermatozoa-oocyte fusion. However, high concentration of seminal ROS is harmful in man (Moustafa *et al*., 2004; Aktan *et al*., 2013), and it alters semen parameters (Agarwal *et al*., 2014; Homa *et al*., 2015). ROS can also produce punctual mutations and polymorphisms, resulting in decreased semen quality (Spiropoulos *et al*., 2002).

Sperm chromatin structure assay (SCSA), terminal deoxynucleotidyl transferase (dUTP) nick-end labeling (TUNEL) assay, Comet assay, Acridine orange test and sperm chromatin dispersion (SCD) are some methods commonly used to identify sperm DNA fragmentation. SCD is characterized by its easy and fast application, as well as, low-cost.

It is based on the halo test, when sperm are treated with an acid solution prior to lysis buffer. The DNA dispersion halos found in sperms nuclei with fragmented DNA after the removal of nuclear proteins are either minimally present or not produced at all. (Fernandez *et al*., 2003; Fernandez *et al*., 2005). Studies have shown that SCD has high sensitivity to detect sperm with DNA fragmentation (Chohan *et al*., 2006; Zhang *et al*., 2010; Liffner *et al*., 2019). Different studies using SCD test have determined the reproductive capacity of the sperm and its correlation with seminal parameters and embryo quality after in vitro fertilization (Velez de La Calle *et al*., 2008; Feijó & Esteves, 2013; Tandara *et al*., 2013; Acosta & Dueñas, 2014; Acosta *et al*., 2015; Borges *et al*., 2019).

The purpose of our study was to determine the correlation between DFI and semen parameter in patients with normozoospermia, oligozoospermia, asthenozoospermia and teratozoospermia using the sperm chromatin dispersion (SCD) test.

## MATERIALS AND METHODS

### Patients

This descriptive retrospective study included 418 patients seen at the Andrology Laboratory of the Assisted Reproduction Center ¨In Vitro Gestar¨, Chiclayo - Perú, between October 2013 and March 2020. We excluded those patients with azoospermia, varicocele, cryptorchidism, antibiotic treatments, diabetes, a history of radiotherapy and chemotherapy, chronic diseases. In Vitro Gestar Committee approved this study.

#### Sample collection and Semen Analysis

The seminal samples were collected by masturbation into a sterile container after a sexual abstinence period of 3 - 7 days. The semen parameters were evaluated according to the WHO guidelines (WHO, 2010). Concentration and sperm motility were determined using a Makler chamber (Sefi-Medical Instruments, Haifa - Israel), and strict Kruger criteria was used for assessing sperm morphology (Papanicolaou staining). To find the normozoospermic patients (NORMO) and patients with an altered parameter (ALTERED), we followed the lower reference limit established by the WHO. The oligozoospermic patients were divided into groups: Severe/Moderate oligozoospermia (OLIGO SM) : <5x10^6^ sperm/mL; mild oligozoospermia (OLIGO M): 5 to <15x10^6^ sperm/mL. The asthenozoospermic patients were divided Based on their progressive motility (PM): Severe asthenozoospermia (ASTHENO S): <10% PM; Moderate asthenozoospermia (ASTHENO MO): 10 to <20% PM; Mild asthenozoospermia (ASTHENO M): 20 to <32% PM. The teratozoospermic patients (TERATO) are those with <4% normal morphology. The oligoasthenoteratozoospermic patients (OAT) are those with <15x10^6^ sperm/m, <32%PM and <4% normal morphology.

#### Determination of DNA fragmentation Index (DFI)

We measured the DNA fragmentation Index of spermatozoa using the Sperm Chromatin Dispersion test (SCD) (Fernández *et al*., 2003; 2005) with the Halosperm Kit (Halotech DNA) with minor modifications. The normozoospermic patients’ seminal samples were diluted in PBS (Phosphate-buffered saline) until reaching a concentration of 5 to 10 x10^6^ sperm/mL, and the oligozoospermic patient’s seminal samples were diluted in 1:1 (vol/vol) in PBS. The eppendorf with agarose (low melting point) was heated for 5 minutes at 90º to 100ºC. Then it was placed in a water bath at 37ºC by 5 minutes. Then, 20µL of diluted semen was added and homogenized. 10µL of this homogenate was placed on a pretreated slide with agarose (normal melting point) and covered with a 22x22mm cover slide. The slides were placed under refrigeration at 4ºC for 7 minutes. Then the cover slides were gently removed and we added the acid solution (20µL HCl added to 2.5mL of distilled water) for 7 minutes. After the removal of this solution, we added the lysis solution for 15 minutes. Then the lysis solution was removed, and the slide was placed in distilled water and alcohol (70%. 90% and 100%) for two minutes each. We left the slide for drying at room temperature, and then we stained it with Wright dye. We analyzed 500 sperm per slide to determine the DNA fragmentation index (DFI). There were five types of halos: big halo, medium halo, small halo, without halo and degraded. Big and medium halo are considered as spermatozoa with normal DNA fragmentation and small halo, without halo and degraded as fragmented DNA spermatozoa.

#### Statistical Analysis

The data was analyzed using the SPSS 22.0 software for windows (SPSS, Chicago, IL, USA). The data is presented as mean ± standard deviation (SD). For the statistical comparison between the different groups, we tested the significance of difference using the Mann-Whitney test and the Kruskal-Wallis test. The regression and correlation analyses were performed using the Spearman's correlation coefficient. The level of significance was set at *p*<0.05.

## RESULTS

The age of the patients ranged from 19 to 59 years with a mean of 38.79±6.91. We analyzed the seminal parameter and DFI of NORMO and ALTERED patients (Table 1). The values of concentration, progressive motility and normal morphology were significantly lower among the ALTERED patients (*p*=0.000). The DFI value was significantly high among the ALTERED patients (*p*=0.003). In addition, the ALTERED patients’ ages were significantly higher when compared to the NORMO patients’ ages (*p*=0.004).

**Table 1:** Descriptive statistics and comparison between NORMO and ALTERED patients

	NORMO n= 132 Mean ± SD	ALTERED n= 286 Mean ± SD	*p* value
Age	37.26 ± 6.76	39.50 ± 6.87	0.004
Volume (mL)	2.85 ± 0.95	2.85 ± 1.27	0.357
Concentration (x106/mL)	76.08 ± 36.36	53.41 ± 39.38	0.000
Progressive motility (%)	47.59 ± 10.60	26.72 ± 15.55	0.000
Normal morphology (%)	4.67 ± 1.05	2.77 ± 1.28	0.000
DFI (%)	15.64 ± 7.65	18.41 ± 9.43	0.003

NORMO: Normozoospermic patients. ALTERED: patients with an altered parameter

In our study, the NORMO patients had significantly higher values (*p*=0.000) in concentration, progressive motility and normal morphology, and a significantly lower value in DFI (*p*=0.003), when compared with other groups with oligozoospermia (Table 2). The same trend occurred in favor of patients with normal concentration, compared with the oligozoospermic group. Although the DFI value is low in normozoospermic patients (NORMO), when compared to patients with normal concentration, there were no significant differences between them. The patients with OLIGO SM had a significantly lower value (*p*=0.000) in concentration, progressive motility and normal morphology; and significantly higher (*p*=0.002) percentage of DFI when compared with the NORMO group (Table 2).

**Table 2:** Descriptive statistic and comparison between group of oligozoospermia patients with normal concentration, and normozoospermic patients

	OLIGOZOOSPERMIA		CONC N n=366	NORMO n=132	*p* value
	OLIGO SM n=11	OLIGO M n=41			
Age	37.09 ± 6.30	38.37 ± 7.08	38.89 ± 6.92	37.26 ± 6.76	0.178
Volume (mL)	2.86 ± 1.54	2.88 ± 1.26	2.85 ± 1.16	2.85 ± 0.95	0.869
Concentration (x106/mL)	2.20 ± 1.46	11.03 ± 2.76	67.88 ± 37.14^a,d^	76.08 ± 36.36^b,c^	0.000
Progressive motility (%)	17.98 ± 12.62	19.47 ± 13.53	35.32 ± 16.73^a,d^	47.59 ± 10.60^b,c^	0.000
Normal morphology (%)	1.82 ± 1.47	2.73 ± 1.23	3.49 ± 1.49^a,d^	4.67 ± 1.05^b,c^	0.000
DFI (%)	26.38 ± 12.94	19.81 ± 11.40	17.01 ± 8.39^a,d^	15.64 ± 7.65^c^	0.003

OLIGO SM: Severe/Moderate oligozoospermia (<5x10^6^/mL). OLIGO M: Mild oligozoospermia (5 to <15x10^6^/mL). CONC N: Normal Concentration (≥15x10^6^/mL). NORMO: Normozoospermic patients.

*p* value between all the groups

a *p*<0.01 between group oligozoospermia (OLIGO SM and oligo M) and normal concentration

b *p*<0.05 between normal concentration and normozoospermic patients.

c *p*<0.005 between OLIGO SM and normozoospermic patients.

d *p*<0.01 between OLIGO SM and normal concentration.

Table 3 shows the comparison of seminal parameters and DFI between the asthenozoospermic and the normozoospermic patients. The NORMO group presents concentration, progressive motility and normal morphology values significantly higher (*p*=0.000) and significantly lower ages (*p*=0.042) and DFI (*p*=0.000); when compared to the other group. In addition, the patients with normal progressive motility had significantly higher concentration, progressive motility and normal morphology (*p*=0.000), and a significantly lower DFI (*p*=0.000), when compared with the group of asthenozoospermic patients. When comparing the NORMO group with patients with normal progressive motility, only the normal morphology group had significant differences (3.76 ± 1.42 vs 4.67 ± 1.65 p = 0.000, respectively). The ASTHENO group patients had significantly lower concentration, progressive motility and normal morphology values, and a significantly low percentage of DFI, when compared with the NORMO patients group.

**Table 3:** Descriptive statistic and comparison between group of asthenozoospermia, normal patients with progressive motility and normozoospermic patients

	ASTHENOZOOSPERMIA			NORMAL MOTILITY n=229	NORMO n=132	*p*
	ASTHENO S n=41	ASTHENO MO n=66	ASTHENO M n=82			
Age	40.20 ± 6.86	39.95 ± 7.34	39.02 ± 7.06	38.12 ± 6.69^d^	37.26 ± 6.76^c^	0.042
Volume (mL)	2.84 ± 1.50	2.67 ± 1.15	2.93 ± 1.31	2.87 ± 1.06	2.85 ± 0.95	0.449
Concentration (x10^6^/mL)	34.11 ± 31.40	41.64 ± 33.83	60.64 ± 47.16	70.74 ± 35.75^a,d^	76.08 ± 36.36^c^	0.000
Progressive Motility (%)	5.75 ± 2.80	15.30 ± 2.92	25.40 ± 3.46	46.27 ± 10.47^a,d^	47.59 ± 10.60^c^	0.000
Normal Morphology (%)	2.22 ± 1.21	2.97 ± 1.36	3.19 ± 1.58	3.76 ± 1.42^a,d^	4.67 ± 1.05^b,c^	0.000
DFI (%)	23.09 ± 11.45	18.78 ± 10.74	17.88 ± 9.56	16.06 ± 7.16^a,d^	15.64 ± 7.65^c^	0.000

ASTHENO S: Severe asthenozoospermia (<10%). ASTHENO MO: Moderate asthenozoospermia (10 to <20%). ASTHENO M: Mild asthenozoospermia (20 to <32%). NORMAL MOTILITY (≥32%). NORMO: Normozoospermic patients.

*p* value between all the groups

a *p*<0.005 between group oligozoospermia (ASTEHNO S, ASTHENO MO and ASTHENO M) and normal progressive motility.

b *p*=0.000 between normal progressive motility and normozoospermic patients.

c *p*<0.01 between ASTHENO S and normozoospermic patients.

d *p*<0.05 between ASTHENO S and normal progressive motility

In the present study, the NORMO group had concentration, progressive motility and normal morphology values significantly higher (*p*=0.000), and significantly lower ages (p=0.001) and DFI (*p*=0.005), when compared with the teratozoospermic patients (Table 4). Although the DFI values were lower in NORMO compared with normal morphology patients, there were no significant differences between them (Table 4).

**Table 4:** Descriptive statistic and comparison between Teratozoospermic patients, patients with normal morphology and normozoospermic patients

	TERATO n=222	NORMAL MORPHOLOGY n=196	NORMO n=132	*p*
Age	39.83 ± 6.77	37.61 ± 6.89^a^	37.26 ± 6.76^c^	0.001
Volume (mL)	2.83 ± 1.23	2.87 ± 1.10	2.85 ± 0.95	0.548
Concentration	51.00 ± 34.34	71.41 ± 42.82^a^	76.08 ± 36.36^c^	0.000
Progressive Motility (%)	28.41 ± 16.48	38.87 ± 16.26^a^	47.59 ± 10.60^b,c^	0.000
Morphology (%)	2.25 ± 0.80	4.64 ± 1.04^a^	4.67 ± 1.05^c^	0.000
DFI (%)	17.96 ± 9.23	17.04 ± 8.71	15.64 ± 7.65^c^	0.053

TERATO: Teratozoospermic patients. NORMO: Normozoospermic patients.

*p* value between all the groups

a *p*<0.005 between teratozoospermic and normal morphology patients.

b *p*=0.000 between normal morphology and normozoospermic patients

c *p*<0.05 value between teratozoospermic and normozoospermic patients.

In table 5, we compared NORMO and oligoasthenoteratozoospermic patients (OAT). DFI was significantly higher in OAT compared with NORMO patients, 22.05±12.15 vs 15.64±7.65, *p*=0.001, respectively.

**Table 5:** Comparison of the DNA fragmentation index (DFI) between normozoospermic and oligoasthenoteratozoospermic (OAT)

	NORMO (n= 132)	OAT (n= 37)	*p*
DFI	15.64 ± 7.65	22.05 ± 12.15	0.001

**Figure 1:**
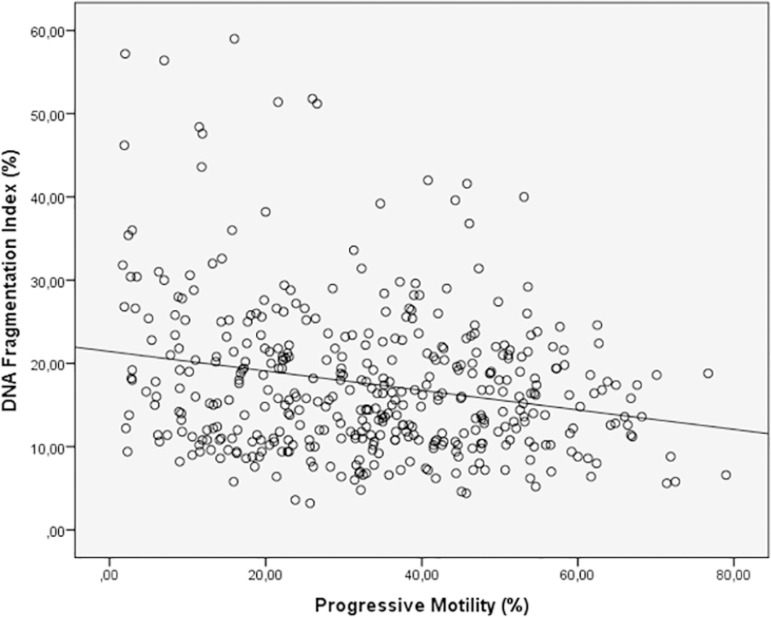
Correlation between Progressive motility and DFI. Individual data points and the regression line are shown. Spearman's correlation coefficient= -0.162 *p*=0.001.

**Figure 2:**
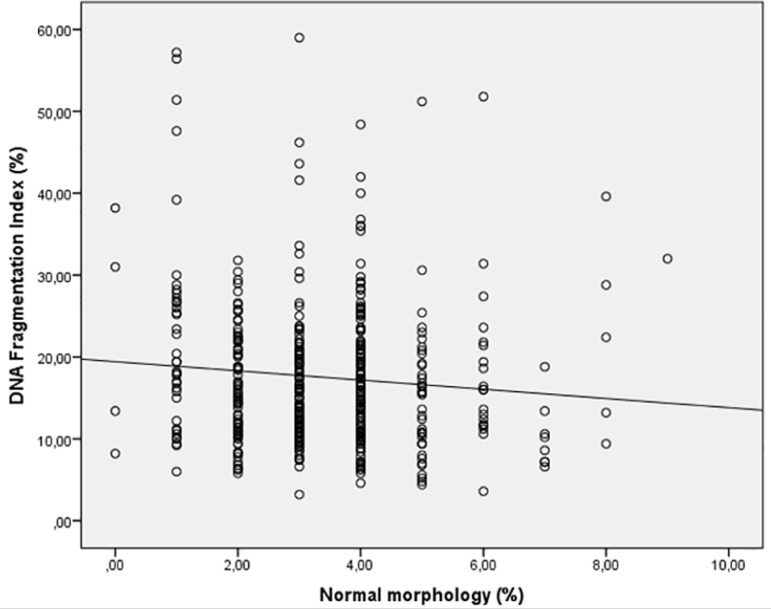
Correlation between Normal morphology and DFI. Individual data points and the regression line are shown. Spearman's correlation coefficient= -0.100 *p*=0.040.

**Figure 3:**
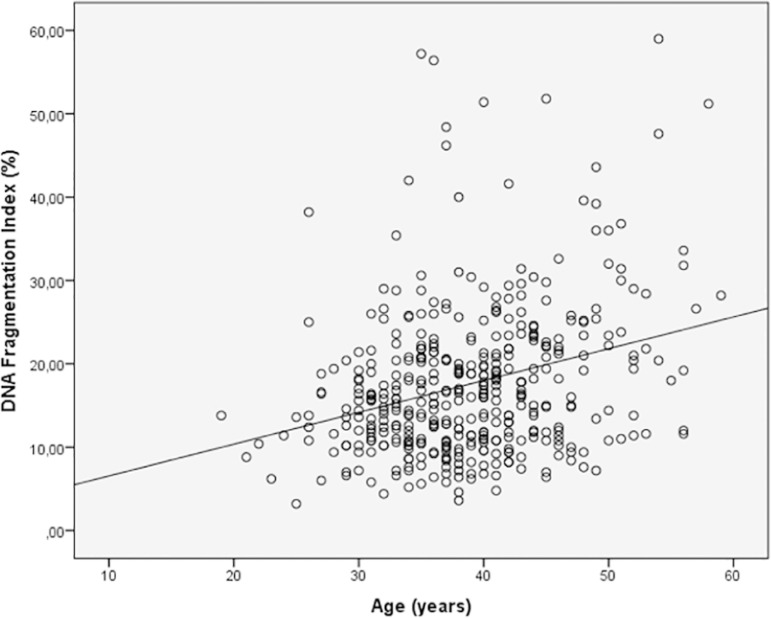
Correlation between age and DFI. Individual data points and the regression line are shown. Spearman's correlation coefficient= 0.257 *p*=0.000.

Spearman's correlation analysis indicated that the progressive motility (r= -0.162 *p*=0.001) (Figure 1) and normal morphology (r= -0.100 *p*=0.040) (Figure 2) had significant negative correlations with DFI. Age (r=0.257 *p*=0.000) had a positive correlation with DFI (Figure 3). We did not find a significant correlation between age and sperm concentration (r= -0.17 *p*=0.732).

## DISCUSSION

The semen conventional analysis (sperm concentration, progressive motility and morphology) is used to assess fertility in men. However, the lack of correlation with the fertilizing potential make necessary to introduce new routine tests, such as sperm DNA fragmentation. High DFI values are correlated with reduced fertilization rates, early development embryo, bad embryo quality, low pregnancy rates and higher rates of spontaneous miscarriage (Bungum *et al*., 2007; Lewis *et al*., 2013; Simon *et al*., 2014). For these reasons, various authors have recommended the introduction of sperm DNA fragmentation analysis as a routine and complementary test in semen analysis (Lewis & Aitken, 2005; Fortunato *et al*., 2013). In this study, we uses the SCD test due its high sensitivity to detect sperm with DNA fragmentation (Chohan *et al*., 2006; Zhang *et al*., 2010; Liffner *et al*., 2019).

This study found that sperm concentration, progressive motility and normal morphology were significantly lower in ALTERED patients, when compared with NORMO patients, while semen volume did not differ (Table 1). Several studies have confirmed the correlation between altered semen parameters and high ROS concentrations (Agarwal *et al*., 2014; Majzoub et al., 2018; 2019); and up to 80% of infertile men have elevated seminal ROS (Agarwal & Sekhon, 2014). Likewise, the current evidence recognizes oxidative stress as an important cause of sperm DNA fragmentation (Aitken & De luliis, 2010; Iommiello *et al*., 2015). Thus, we believe that high DFI values among the ALTERED patients is due to high concentrations of ROS produced by decreased semen quality in these patients. These results are consistent with other studies from Peru, such as those of Acosta & Dueñas (2014), which found a mean DFI value among ALTERED patients to be significantly higher than in the NORMO patients (22.95 ± 12.25 vs 14.39 ± 9.06); and Acosta *et al*. (2016) found significant differences in the mean value of DFI between ALTERED and NORMO patients (21.51 ± 14.18 vs 14.08 ± 7.08, respectively). Similar results were reported by other studies evaluating fertile and infertile patients using the SCD test (Fernández *et al*., 2005; Chohan *et al*., 2006; Evgeni *et al*., 2015; Wiweko & Utami, 2017; Majzoub *et al*., 2019) and other tests (Sergerie *et al*., 2005; Chohan *et al*., 2006; Sheikh *et al*., 2008; Mayorga-Torres *et al*., 2013; Di Santo *et al*., 2016). On the other hand, the study by Khalili *et al*. (2006) did not find significant differences in the value of DFI between fertile and infertile patients using the acridine orange staining test. We believe that the lack of correlation with DFI in this study is due to the assay used. The acridine orange staining assay is questionable, and is not recommended as a screening test for sperm quality and functional capacity due its very low clinical significance for infertility testing, and lack of relationship with other test, such as SCSA, TUNEL and SCD (Chohan *et al*., 2006).

We found that age has a high significance in ALTERED patients when compared with NORMO patients (39.50 ± 6.87 vs 37.26 ± 6.76, respectively) (Table 1). Male aging is associated with infertility, semen altered parameters and sperm DNA fragmentation. Aging causes accumulation of ROS, leading to increased oxidative stress that induces lipid peroxidation and further ROS generation in the mitochondria (Gunes *et al*., 2016). An excessive amount of ROS and decreased antioxidant capacity in the course of aging may induce apoptosis or oxidative damage to DNA. Our results are consistent with the study from Majzoub *et al*. (2019), and discordant with the studies from Acosta & Dueñas (2014) and Acosta *et al.* (2016).

Our study shows significant differences in the DFI between normozoospermic and oligozoospermic patients (Table 2). Our data is congruent to several studies (Varshini *et al*., 2011; Aydos *et al*., 2015; Choucair *et al*., 2016). A possible explanation for this relationship is the increased apoptosis of mature spermatozoa. Apoptosis controls the overproduction of male gametes (Sakkas *et al*., 1999). Increased levels of ROS are correlated with increased apoptosis of mature spermatozoa. (Agarwal & Sekhon, 2011). Oxidative stress-induced DNA damage may accelerate the process of germ cell apoptosis, leading to a decline in sperm count (Agarwal *et al*., 2003).

Progressive motility is one of the important parameters involved in sperm fertilizing capacity. This study shows DFI significant differs between normozoospermic patients and the asthenozoospermic group (Table 3). Our results are consistent with several studies. Belloc *et al*. (2014) determined that sperm motility is the seminal parameter that is most intimately related to sperm DNA fragmentation. In their study, they found a significant high level of DFI in males with asthenozoospermia alone when compared to oligozoospermia, or isolated teratozoospermia. Elbashir *et al*. (2018) found a significant negative correlation between DFI and progressive motility between infertile asthenozoospermic men and men with proven fertility. On the other hand, Varshini *et al*. (2011) reported a statically non-significant difference in DFI using TUNEL between asthenozoospermic patients and normozoospermic patients, despite having high median distribution. A possible explanation of the association between DFI and asthenozoospermia can be found in the development of the flagellum in the spermatogenesis. Cho *et al*. (2001) showed that the DNA compaction (using protamine or transition protein insufficiency models) is associated with development of an abnormal flagellum and defective motility. Another reason that explains this correlation, may be due to the increased oxidative stress causing sperm DNA damage, and in turn induce the lipid peroxidation of the sperm membrane, which results in oxidation of polyunsaturated fatty acids in the plasma membrane and the formation of malondialdehyde (MDA), thereby leading to structural and functional damage to the spermatozoa (Agarwal & Said, 2005). High levels of MDA is correlated with high DFI, both having a negative correlation with the progressive motility (Dorostghoal *et al*., 2016). Thus, high concentration of ROS can cause decreased sperm motility due to the damage to the axonemal structure or the reduction in intracellular adenosine triphosphate (Tsunoda *et al*., 2012). The decreased sperm motility has also been explained by apoptosis. Oxidative stress causes the generation of spermatozoa with poorly remodelled chromatin. These defective cells have a tendency to enter in an apoptotic pathway associated with motility loss (Aitken & Koppers, 2011).

Teratozoospermia presents a high phenotypic variability and limited information is available about its pathophysiological mechanism. Morphological anomalies of the spermatozoa play a very important role in determining the male fertility potential. DNA damage is correlated with different abnormal shape of the head and flagellum of sperms, being the anomaly of the head that presents a highly significant DFI value (Eskandari *et al*., 2017). In this study, we found a significant DFI correlation between teratozoospermic and normozoospermic patients (Table 4). Previous studies have demonstrated a positive correlation between teratozoospermia and DFI (Varshini *et al*., 2011; Garcia-Ferreyra *et al*., 2014; Aydos *et al*., 2015); and other studies have not reported a significant relationship between these parameters (Avendaño *et al*., 2009; Choucair *et al*., 2016). One of the processes that explains this relationship is the incomplete replacement of histone by protamine, which induces abnormal chromatin condensation, producing deformations of the nucleus and overall head shape in the sperm (Ma *et al*., 2019). Another explanation may be the abnormal apoptosis causing oxidative stress. The abnormal apoptosis may cause the persistence of abnormal spermatozoa that are marked for elimination, thereby increasing teratozoospermia (Sakkas *et al*., 1999). Aydos *et al*. (2015) determined that the positive correlation between sperm DNA damage and impaired sperm morphology might be associated with the fact that sperm DNA damage leads to impairment in the sperm chromatin structure.

Our results indicate that DFI is significantly higher in men with OAT when compared with normozoospermic men (Table 5). Several studies have investigated the association between DFI and conventional seminal parameters. Most of these studies are consistent with our results (Varshini *et al*., 2011, Acosta *et al*., 2015; Aydos *et al*., 2015; Choucair *et al*., 2016), confirming that male infertility is associated with poor sperm DNA integrity.

The present data demonstrated a statically significant inverse correlation between the DFI percentage, sperm progressive motility and sperm normal morphology (Figure 1, 2). We did not find a significant correlation with sperm concentration. Many studies have reported a significant negative correlation between DFI and semen parameters, mainly concentration (Velez de la Calle *et al*., 2008; Acosta & Dueñas, 2014; Aydos *et al*., 2015; Acosta *et al*., 2015; Choucair *et al*; 2016), progressive motility (Sheikh *et al*., 2008; Velez de la Calle *et al*., 2008; Acosta & Dueñas, 2014; Acosta *et al*; 2015; Aydos *et al*., 2015; Evgeni *et al*., 2015; Choucair *et al*; 2016; Elbashir *et al*., 2018) and normal morphology (Velez de la Calle *et al*., 2008; Fortunato *et al*., 2013; Acosta & Dueñas, 2014; García-Ferreyra *et al*., 2014; Aydos *et al*., 2015), alone or in combination. On the other hand, the study by Khalili *et al*. (2006) did not find correlations between DFI and sperm concentration, progressive motility and normal morphology using the acridine orange staining test. Likewise, we found a significant positive correlation between DFI and age, but this is considered a weak correlation (r = 0.257) (Figure 3). These correlations are similar to those reported by other authors: Acosta & Dueñas (2014) r = 0.198 *p* = 0.009, García-Ferreyra *et al*. (2014) r = 0.106 *p* = 0.0001, Acosta *et al*. (2015) r = 0.198 *p* = 0.009 and Petersen *et al*. (2018) r = 0.14 *p* = 0.002. Other studies found a strong correlation between sperm DNA fragmentation and age; Plastira *et al*. (2007) r = 0.558 *p* <0.001, and other authors did not find significant correlations (Winkle *et al*., 2009; Brahem *et al*., 2011). Petersen *et al*. (2018) concluded that unfortunately not all studies follow these statistical analyzes, which makes it difficult to interpret this data.

In conclusion, our results suggest that an abnormal spermogram not only reflects altered spermatogenesis but also a negative effect on sperm DNA, and high DFI is accompanied by significant impairment to all seminal parameters.
